# Understanding the Social Meaning of Infertility and Childbearing: A Qualitative Study of the Perception of Childbearing and Childlessness in Northern Ghana

**DOI:** 10.1371/journal.pone.0054429

**Published:** 2013-01-16

**Authors:** Philip Teg-Nefaah Tabong, Philip Baba Adongo

**Affiliations:** 1 Ghana Health Service, Brong Ahafo Regional Hospital, Sunyani, Ghana; 2 Department of Social and Behavioural Science, School of Public Health, College of Health Sciences, University of Ghana, Legon, Ghana; McGill University AIDS Centre, Canada

## Abstract

**Background:**

Infertility is a major medical condition that affects many married couples in sub-Saharan African and as such associated with several social meanings. This study therefore explored community's perception of childbearing and childlessness in Northern Ghana using the Upper West Region as a case study.

**Methods:**

The study was exploratory and qualitative using in-depth and key informant interviews and focus group discussions. Fifteen marriage unions with infertility (childless), forty-five couples with children, and eight key informants were purposively sampled and interviewed using a semi-structured interview guides. Three focus group discussions were also carried out, one for childless women, one for women with children and one with men with children. The data collected were transcribed, coded, arranged, and analyzed for categories and themes and finally triangulated.

**Results:**

The study revealed that infertility was caused by both social and biological factors. Socially couples could become infertile through supernatural causes such as bewitchment, and disobediences of social norms. Abortion, masturbation and use of contraceptives were also identified as causes of infertility. Most childless couples seek treatment from spiritualist, traditional healers and hospital. These sources of treatment are used simultaneously.

**Conclusion:**

Childbearing is highly valued in the community and Childlessness is highly engendered, and stigmatised in this community with manifold social consequences. In such a community therefore, the concept of reproductive choice must encompass policies that make it possible for couples to aspire to have the number of children they wish.

## Introduction

Infertility or childlessness is a global reproductive issue for both sexes yet often neglected and not discussed in public. It is generally believed that more than 70 million couples suffer from infertility worldwide [1]. In Sub-Saharan Africa, the prevalence differs widely from 9% in the Gambia [2], 21.2% in northwestern Ethiopia [3], between 20 and 30% in Nigeria [4], [5] and 11.8% among women and 15.8% among men in Ghana [6]. There are varying opinions on the definition of infertility. The lack of consensus on the prevalence of infertility is a consequence of differing definitions of infertility, the varying periods of time over which it is studied, and a failure to differentiate analytically between voluntarily childless and involuntarily childless [7]. However, the Ghana Demographic Health Survey reports that voluntary childlessness is not common in Ghana, and currently married women with no live births are likely to be those in relationships with fertility problems [8]. Infertility has been defined as failure to conceive after one year of regular unprotected sexual intercourse in the absence of known reproductive pathology [9]. However, epidemiological studies have revealed that in a normal population of heterosexually active women who are not using birth control methods, 25% will become pregnant in the first month, 63% within 6 months, and 80% within one year. By the end of a second year, 85% to 90% will have conceived [10]. Because some couples, who are not infertile, may not be able to conceive within the first year of unprotected sex, the World Health Organisation therefore recommends the epidemiological definition of infertility, which is the inability to conceive within two years of exposure to pregnancy [9]. Infertility may be primary or secondary. Primary infertility refers to infertility of women who have never conceived and secondary infertility refers to infertility of women who has conceived at least once before. The use of the ability of the female to conceive as a measure to differentiate between primary and secondary infertility is however problematic as it places couple infertility on the doorsteps of the female partners.

In the African culture, the true meaning of marriage is only fulfilled if the couple conceives and bears children [11]. Africans consider their child to be a source of power and pride, and children act as insurance for their parents in old age. The most important aspect of bearing children is an assurance of family continuity. Anthropological and sociological studies bear testimony to the considerable suffering associated with involuntary childlessness due to negative psychosocial consequences such as marital instability, abuse and stigmatization [11], [12]. A study among women seeking infertility treatment in Southern Ghana revealed that infertile women used their internal coping strategies by keeping their fertility problem to themselves as a result of the stigma associated with it whilst others coped by drawing on their Christian faith [13].

There are varying findings on the contribution of the various sexes to couple infertility. An increasing body of social science and biomedical evidence suggests that nearly 40–50% of infertility is attributable to problems suffered by men. The underlying cause of infertility may be a male factor (40%), a female factor (40%) or a combination (20%) of problems [14]. Another study states that about 50% of infertility is due to the female, 20–26% to the male and 26–30% is unexplained (couple factor infertility was not reviewed), but these findings are all from non-African populations [6]. Studies have revealed that the most common cause of male infertility is due to a problem in the sperm production process in the testes. About two thirds of infertile men have sperm production problems. Low numbers of sperm are made and/or the sperm that are made do not work properly [15]. A number of factors can disrupt the production of sperm including undescended testis, infections such as mumps, heat, sperm antibodies, torsion, varicocele, drugs or radiation damage. Blockages (obstructions) in the tubes leading sperm away from the testes to the penis can cause a complete lack of sperm in the ejaculated semen. This accordingly is the second most common cause of male infertility and affects about three in every twenty infertile men, including men with the common problem of having an earlier vasectomy [15]. In some men, sperm antibodies can develop which can lessen sperm movement and block egg binding during fertilisation. About one in every sixteen infertile men has sperm antibodies [16] and this may cause male infertility.

In women, poor quality eggs may cause infertility, a blocked fallopian tube could prevent the egg and sperm from uniting, or the woman may not ovulate regularly – a problem that is sometimes results in irregular menstrual cycle [17]. There is a well-established link between a woman's age and infertility. Women over age 35 years have an estimated 50 percent chance of becoming pregnant naturally [16]. As women age, their fertility is affected by the quantity and quality of their eggs. In reality, the number of eggs available in the ovary gradually declines. As menopause approaches, an increasing number of cycles are not ovulatory, and therefore unable to result in conception [17]. Moreover, an older woman's eggs are most susceptible to chromosomal changes that may produce abnormal embryos [18]. A study in Bawku in Northern Ghana identified tubal damage, male factor, anovulation, uterine factors and unexplained as the five main causes of infertility. This study also states that about 20% of infertility in Bawku in Northern Ghana was due to male factors [19].

In spite of these statistics, women still endure the worst of the blame for infertility problems. Leading male Obstetricians and Gynaecologists are often quick to attribute couple infertility to female factors and can therefore be blamed for the belief that is widely held [20]. Infertility is socially constructed in many cultures that is, men and women are meant to become parents and that women are especially socialized to become mothers [21]. Infertility is a problem both medically and socially in Ghana. Medically because there are reports of high prevalence of infertility among couples with inaccessible fertility services. Infertility is also a problem socially because we live in a society where womanhood and manhood are generally tied to motherhood and fatherhood respectively. Despite these problems of infertility, not much has been done to understand community's perception of infertility and childbearing in Ghana. It is in the light of this that this study examines community perception and understanding of childbearing and childlessness in Northern Ghana.

## Materials and Methods

### Ethics Statement

Ethical approval for the study was received from the Ghana Health Service Ethics Committee. During the approval process for the study, the committee was explicit about the need to maintain confidentiality and anonymity whilst emphasising on the need to obtain verbal or written consents. In line with the approved procedure of obtaining consent for the study, verbal or written consent were obtained from participants. Verbal consent was obtained from participants who had difficulty reading the consent form and those who opted to give verbal consent. To those who gave verbal consent, the researchers read and translated the consent form into the preferred language of the participant. They were further made to recommend a neutral member of the household to act as independent witness in the consent process. A cover sheet containing the demographic information except the names and locations that were coded and kept separately was used to document those who gave verbal consent. This procedure for obtaining verbal consent was approved by the Ethics Committee of the Ghana Health Service. To ensure confidentiality of participants that gave written consents, codes were used on the form instead of their names. The specific locations of the participants were also not reported as this could lead to easy identification of the infertile couples that took part in the study. In addition, only codes were written on interview transcripts.

### Study Area

The Upper West Region is the smallest Region in Ghana with a population of 702,110 with 989 settlements. The region covers a total land area of 18,476 km^2^, with a population density of 32 persons per square kilometre [22]. The region is divided into eleven administrative districts and the people speak three main local languages namely: Wali, Dagaare or Sisali. The people of Upper West Region practice patrilineal mode of inheritance and is a typical patriarchal society with male dominance in decision-making. Polygamy is a common practice both by members of the Islamic community and those who profess African Traditional Religion. In all, there are sixty-five sub-districts, five district hospitals located in four districts, a regional hospital in the regional capital. The region has a fertility rate that is the average number of children per couple of five, which is above the national average of four [22].

### Data Collection

In-depth interviews (IDI), Focus Group Discussions (FGD) and Key Informant Interviews (KII) were the main data collection methods used in the study. Four (4) trained data collectors (Research Assistants) were used in the study. These research assistants were put in groups of two, a male and a female. Male interviewers were assigned for male respondents and female interviewers were assigned for female respondents with children. Feminist theorists argue that the positionality of the researcher: gender, class, and race affect all aspects of the research process, from the framing of the research question to the analytical approach as one's own social location influences the full scale of research choices [23]. In-depth interviews are social interactions [24] and hence race, class, and gender inequalities are inherent in these interactions and can therefore affect the results. Hence, interviewer/interviewee homogeneity was adopted to overcome this challenge of the effects of differences in gender between the interviewer and interviewee. Concerning couples with infertility (childless), the principal researcher who supervised the data collection carried out the interviews for both partners. This was done because it was anticipated that some respondents might break down emotionally in an attempt to narrate unpleasant past experiences. The principal researcher's background in nursing and psychology therefore put him in a better position to offer counseling for those who break down in the course of the interviews. Interviews were either conducted in English or local language depending on the language the respondent was comfortable with and the duration of the interviews was between 30–45 minutes.

The interviews were tape-recorded with the permission of the participants as well as note taking. In polygamous family, the male's partner and the individual wives were interviewed starting from the most senior of the wives in line with community's norms. Younger wives in some instances were interviewed first upon the request of the most senior wife in consultation with the husband. The same codes were written on the male and female interview guides and each recording was started by first mentioning the code on the interview guide to ensure data collected could be analysed as belonging to a couple for comparison to be made. The codes also differentiated between the male and female partners that constituted a couple.

Field notes were written immediately after each interview. The field notes covered the initial reactions to the interview, including the first analytical reflections from the interview content, and any useful observations that would not be captured by digital recording. This covered the demeanor of the respondents, his or her body language and mood, and any informal conversation that took place before or after the interview.

### Selection of Study Participants

An informal discussion with the community members and Community Health Volunteers (CHVs) was initially used to identify couples without children. After interviewing such couples, snowball techniques were then used to identify other couples with infertility in that community. The study considered a married union as a unit of analysis irrespective of the number of partners involved. Hence, fifteen (15) units of childless marriages were interviewed. However, three of the childless male partners in the study were married to two wives. This therefore increased the number of childless individuals in the study to thirty-three (33) comprising of fifteen (15) childless males and eighteen (18) childless females. The age for the males' partners ranged between 35–63 years as against a female age range of 28–52 years. The couples were married for between 3–25 years and included couples with both primary and secondary infertility.

Couples with children were also purposely selected and interviewed. The couples were recruited from both urban and rural settlements where childless couples lived. In all, forty-five (45) married unions were recruited and interviewed within the neighbourhood (households) where couples without children lived. However, four of the males were married to two wives whilst one was married to three wives increasing the number of women with children interviewed to fifty-one (51) with ninety-six (96) participants with children. The daily analysis of the transcripts made the research saturated with Forty-five married unions. Collecting enough data to the point of saturation adds to the credibility, dependability of the study and transferability of the study results to other studies on infertility and childbearing.

### Focus Group Discussion

Three Focus Group Discussions (FGDs) were organized. One FGD for women without children as their male counterpart refused to take part in FGD. Their refusal to take part in the FGD was because infertility was perceived to be the inability of women to bear children and therefore not suitable for males to discuss. The researchers organized two (2) FGDs for community members selected from where infertile couples were resident, one for males and one for females to elicit normative ideas on childbearing and childlessness in the communities. The discussants drawn from the communities were all married adults with children. The FGDs lasted for between 60–90 minutes and were conducted in the evenings as participants preferred to do that after the day's work. All Focus Group Discussions were conducted in Dagaare (local dialect) however; participants who wished to contribute in English at any point in the discussions were allowed to do that. Such contributions were translated immediately to Dagaare for participants who did not understand English. This was done to ensure that such contributions conformed to the normal practice in the community. All participants were allowed to give their view on any subject raised before progressing to another theme.

### Key Informant Interviews (KII)

Eight (8) Key Informants Interviews (KIIs) were conducted. Two Gynaecologists who take care of infertile couples were interviewed as Key Informants. An Islamic scholar was also interviewed to provide expert view on infertility and childbearing in the Islamic Community. A female Christian leader was also interviewed as she provided support for infertile females. Two traditional medical practitioners who also provided care for infertile couples were further interviewed. A manager of the National Health Insurance Scheme and a manager with Private Insurance Company were equally interviewed as Key Informants to provide information on the infertility related insurance policies in Ghana.

### Data Analysis

The taped interviews were transcribed verbatim and the resulting texts analysed by using thematic analysis. An attempt was first made to extract broad themes from the transcripts and then progressed to identifying coded themes. In establishing themes, considerations were given to statements of meaning that were present in most of the relevant data. In an attempt to ensure, the credibility of the findings independent coders were used to verify or corroborate the themes extracted from the data. This allowed the researchers to progressively focus the interviews and observations, and to decide how to test the emerging conclusions. Individual and comparative analysis of the response of couples was carried out. The transcripts were entered into QSR Nvivo 8© for analysis. We developed a codebook based on the major themes of the study. The major themes were transformed in tree nodes and free nodes. The authors based on the codebook developed and verified independently coded texts from the transcriptions. The emerging themes and sub-themes were identified and written out in the results. Quotes from respondents were used to support the emerging patterns of concepts from the data.

## Results

### Reasons for Preference for Children

The pinnacle of reasons for preference for children was to maintain the family lineage and inheritances. Children are often given the family name of the father as the communities practice patrilineal mode of inheritance and they are supposed to marry in future and name their children after the family name. This according to respondents was done to ensure the perpetual existence of the family lineage. Examples were given of great families whose lineage had wiped out because they were unable to give birth to more children and majority gave birth to female children who got married and named their children after their husbands. Life without children was perceived not to be worth living as there will be nobody to inherit the properties of the deceased and not all efforts by such individuals are ever appreciated in the community.

*“Children are supposed to maintain lineage and inherit your property…we are suffering on earth because of our children”* – (A 66-year old man with children in FGD).

A second reason for procreation was for assistance at home and in the farm. The number of children most especially male children is an indication of the worth of the couple as it implies more hands in the farm. More hands in the farm means more revenue for the family as extra farm produce could be sold to generate income for the family. This further means more savings and security for future. This practice has become necessary especially as discussants stated, the break in the extended family system and increasing nucleation of families has made the quest for own children very significant as they provide security in old age.

*“I use to plough only 5 acres of land but with my 5 children, I am now able to plough over 15 acres improving on the family income”*- (A 56-year old man in IDI).

The third reason for procreation, which was stated emphatically by the respondents, was to obey Gods words as the Bible and Koran enjoin Christians and Moslems to multiply and fill the earth. Respondents especially believed that failure to beget children was against the holy books (Bible and Koran) and such a family will never receive the blessings of God.

*“The bible says in genesis that we should multiply and fill the earth to ensure the continuous existence of the earth”*- (A 36-year old man in FGD).

Children are also source of joy, companionship and respect for community members. Children console their parents and are a symbol of achievement for couples. At old age, grandchildren act as companions for grandparents and this was believed to make them happy and prolong their life.

*“Children make you happy, it is the greatest achievement on earth…we respect people with children in this community especially male children”*- (A FGD female participant).

Clearly from the respondents, one of the reasons for procreation is to have a befitting funeral, as there are significant difference in the performance of funeral rituals for people with children and those without children. Adult without children are more likely to be interred earlier in traditional communities and their funeral not attracting the needed attention it deserves as compared to adults with children. This is even more distinct for females than males without children. This is because of women traditional role to beget children to the family of the in-law. This reproductive role becomes the exclusive right of the husband following the acceptance of the bride wealth and not having biological children implies that the woman has failed in a fundamental way resulting in a loss to the family of the husband. In addition, childbirth is the culmination of a woman's rites of passage to adult womanhood. Without a live birth, she remains in an infinitesimal state where she is neither a man nor fully a woman. The rituals performed for a deceased adult without children were comparable to that of a child.

*“Adults without children do not have funerals in this community as they are treated the same way as the death of a child”*- (An FGD male participant).

### Knowledge on Causes of Infertility

According to respondents, infertility was caused by both biological and social factors. The biological causes were more pronounced among the urban and educated residents whilst the rural communities attributed infertility principally to social factors. The highest biological factor that has been blamed for infertility among females was previous use of contraceptives. This was also directly attributed to past promiscuous lifestyle of the woman as the contraceptive were used to prevent unwanted pregnancies.

*“Women who were prostitutes during their youthful ages, used contraceptives to prevent pregnancy…they are the people who by all means become infertile in future and worry their partners. I know of a friend who used those family planning methods when we were growing up and is now hopping from hospital to hospital looking for a child”*- (A 45-year woman in FGD).

The believe in contraceptives as a cause of infertility was unanimous for both male and female participants and couples strongly believed the use of contraceptive could result in them becoming infertile. Some infertile women even attributed and strongly believed that they were currently having difficulty in becoming pregnant because they had used contraceptives in the past to prevent unwanted pregnancies since abortion was illegal in the country. Males in FGD alluded to this belief and indicated that contraceptives were a major enemy in the community because it (contraceptives) use encourages promiscuous lifestyles for the youth and give trouble to their male partners in future.

*“My wife made a mistake and took those drugs (contraceptive), and it took her several years to become pregnant again. This almost resulted in a divorce but I have since warned her never to take those drugs again”*- (A 45-year old man in FGD).

Sexually transmitted infections (STIs), blocked fallopian tubes and uterine fibroid were also mentioned as female factors that can cause infertility. Knowledge on the relationship between STIs and blocked tubes was high as it was unanimously agreed in FGD and cited in in-depth interviews. Participant mentioned gonorrhoea, syphilis and Chlamydia infections as common causes of infertility in the community.

*“My wife was told that her tubes were blocked and that was why we could not get a child, when I heard this I immediately suspected she has had gonorrhoea before”*- (A 42-year old infertile man in IDI).

Abortions in all forms (safe and unsafe) are also believed to cause infertility but most especially those conducted by unqualified individuals. To community members, all women are born with a fixed number of children to conceive and when these children are aborted, and then the consequential effect is infertility. However, males are believed not have such fixed number of children and are therefore capable of producing uncountable number of children. Safe abortion in this context refers to an abortion that has been carried out by a qualified person using appropriate equipment and in a place designed for such procedures. Unsafe abortion, which they believe, is what is performed by quacks in the community and the use of herbs.

*“Some women throw all the children they were supposed to give birth into the gutters through abortions”*- (An FGD female participant).

There was a paradox concerning Female Genital Mutilation (FGM) and infertility. As FGM was mentioned as capable of causing female infertility, an “extra-germination” of the clitoris was also believed to cause infertility. As to which size of the clitoris was considered normal, there was no consensus but it was generally believed that the gods give direction when consulted by the healers. Therefore, the gods will often determine the “extra-germination” in which case it has to be excised by the traditional healer after which herbs are applied.

*“Extra-germination of the clitoris can cause infertility and in such a case you cut off that extra germination”*- (An FGD male participant).

*“….My wife bled highly when a part of her clitoris was excised by a traditional medical practitioner as treatment for infertility”*- (A 46-year childless man in IDI).

Contrary to the female factors that were spontaneously mentioned in IDIs and FGDs, male factors were not mentioned until when prompted by the researcher. Males especially attributed infertility to lifestyles such as intake of excessive alcohol and smoking, though taking of alcohol was perceived to be socially acceptable, smoking was generally believed to be alien to the culture and described as Western culture.

*“Too much intake of alcohol and smoking can cause infertility….It leads to weak penis and impotence”*- (An FGD male participant).

There were varying views on which alcoholic beverages and what amounts could be described as too much. As some participants stated that all alcoholic beverages could cause infertility when not taken in moderation, other believed that bottled drinks were more accountable whilst the local alcoholic beverage *pito* was even considered to increase both sperm production and potency and could be used to treat infertility.

*“Pito can increase sperm production and that is why it is sometimes required that you add pito to the herbs in the management of infertility”*-(A traditional medical practitioner in FGD).

Another school of thought was that bottled alcoholic beverages that are believed to enhance sex and increase appetite could cause infertility. Calls were even made to ban the production and advertisement of all such alcoholic beverages as they have become so common and easily accessible even by minors, participant emphasised.

*“This bottled drinks that can increase erection can cause low sperm production and cause infertility. Any man who relies on drinks to perform will not be able to impregnate his wife or give birth to weak children”*- (A 56-year old man in FGD).

*“You see, our ancestors did not have a problem with childbearing, because they took things that were indigenous, but what do we see today, different varieties of foreign foods in our markets that we are blindly eating….These are responsible for some of the funny conditions we are seeing today”*- (A 48-year man in FGD).

Female participants however mentioned watery sperm and inability of a male to sustain an erection as common causes of male infertility. References were also made to the use of aphrodisiac preparations as they can render a man impotent.

*“Some males produce watery sperms and their penis is usually not strong enough during sex to impregnate a woman that is why we describe such people as having a dead penis”*- (Female infertile FGD participant).

The belief in supernatural (social) causes of infertility was widespread and consistent. The belief that some women are witches, and that either curses can be placed on them or their associates was consistent. Such women are believed to live for longer years because they are capable of exchanging their death with the death of children in the community. Such old women are abandoned by their relatives and hooted at when seen in public and are not allowed to come close to children, as they are believed to be capable of bewitching them. Children are specially socialised to run away upon seeing such old women in the communities and some of them are beaten mercilessly sometimes when seen in public gatherings.

*“A childless woman after killing all the children in her womb recently took the head of a child in my community and was beaten until she returned the head. She has subsequently been banished from the community”*- (An FGD female participant).

Though participants also agreed that some men could be wizards and curses invoked on them, the penalties for such infractions with the gods appear not to include infertility for men. However, there was consensus on wizards also bewitching children but it was a common phenomenon with females.

*“Men who are wizards at old age use their witchcraft to protect their family”*-(An FGD female participant).

One of the main and common social causes was described as a pledge made to the gods by people to sacrifice their manhood or womanhood for wealth referred to as *plumma or donpuli.* This pledge can only be reversed by undergoing some rituals, which is often prescribed by the soothsayers or traditional medical practitioners. However, the fear of losing one's properties and becoming impoverish discourages people from reversing such a pledge. To be able to maintain the wealth and bear children, the individual has to perform several rituals which are always very expensive and not within the ability of infertile couples. Such people are reported to be exploited by healers.

*“Some people exchange their children with wealth but after coming to earth to see people having children, they become interested in children”*- (A 56-year old traditional medical practitioner in FGD).

*“I have sacrificed several animals in an attempt to reverse a pledge I was told I made to the gods to be rich yet the traditional healer is still demanding more because he has seen that am desperate to have a child”*-(A 38-year old childless man in IDI).

Breaking the codes of marriage was also widely held belief as a cause of infertility. The gods and ancestors were believed to be “supernatural policemen” who are capable of rewarding couples with children and punishing those who break the codes of marriage or visiting them with infertility.

*“The gods and ancestors who are the custodians of this land can show their disapproval of the conduct of the marriage couple by making them infertile”*- (An FGD male participant).

Male couples who are currently not seeking biomedical management tended to attribute their infertility to female factors whilst their females' counterparts also blamed the males for their inability to make them pregnant. This was a basis for one to prove his or her fertility by engaging in multiple sex with other partners with the hope of either becoming pregnant or making another woman pregnant. In a response to a question on why it was difficult for them (couple to have children). The female respondent in an individual interview stated:

*“My husband is unable to make me pregnant, for me am fertile; my mother gave birth to eight of us so how can I be infertile”*- (A 36-year old childless woman in IDI).

The male partner of this childless female also stated in an IDI, *“I think my wife is barren because I have made a girl pregnant before when we were growing up”.*

Another supernatural cause of infertility, which was widely reported and justified by Christians was masturbation, which was described as “male abortion”. Masturbation was perceived to be sexual immorality and a sin. Respondents generally believed that masturbation could attract a punishment of infertility as it is deemed an immoral act. Participants believed that masturbation was a form of abortion (male abortion) as sperms were believed to be pre-formed babies that are put in woman's womb to incubate until the woman brings it out during delivery. There was therefore no distinction between a woman aborting a fertilized ovum and a man masturbating and discharging spermatozoa.

*“Sperm are human beings and should not be masturbated and discarded…in fact it is male abortion and God is against abortion”*- (A 45-year old Christian participant in FGD).

### Infertility and Sex Preference

Concerning the definition of infertility, three main themes emerged in interviews and FGDs each without time limit. One related to the medical definition and the others related to the desire to have many children and sex preferences. To the participants, infertility is not only defined as the inability of a couple to beget children. It also includes inability to beget male children or inability to comply with society's norm of having many children. The ideal numbers being a function of the desire of the couples but in many cases about five is preferred.

*“Infertility is the inability to give birth to the number of children that you prefer and most especially male children”*- (A 56-year old FGD participant).

Generally, it is believed that it is the responsibility of the male to maintain the lineage of the family. Hence, male partners will go to all extent to have male children including marrying multiple wives or continue to give birth until such a time that a male child is born. The significance of male children is so intense that couples without male children are treated in the same way as those who do have children at all.

*“I am married to three wives because I thought my first two wives were unable to give me male children. When my third wife gave birth recently, a friend came to congratulate me, after which he asked whether this time my wife had given birth to a human being. With my so many female children, I will be treated like someone who has not given birth at all when I die”*- (A 63-year-old man in IDI).

However, the three wives of the man believed that they were only incubators and therefore only brought out what the man has put in there during delivery and could not be blamed for their inability to give birth to male children.

*“I told my husband that he cannot give birth to male children but he refused and went in for a second and third wife, but see we are all giving birth to females”* -(First wife of a man who is married to multiple wives in IDI).

This is because it is the responsibility of deceased's male child to go to the bush to harvest a special stick known as *Kpiendaa*, which is to be kept in a special room reserved for ancestor to symbolize the deceased acceptance in the ancestral world.

### Help-Seeking Behaviour of Infertile Couples

A consensus reached by the individual interviews and focus group participants was that the treatment of infertility in the community is usually directed specifically at women and that most people use three treatment outlets: churches (spiritualists), traditional healers and hospitals (biomedical). However, there was no agreement between and within the groups on which of the three methods that people prefer most. Nonetheless, there was a strong sense that people often use the three treatment methods in combination and in sequence. The first method chosen is often determined by the perception of the couple regarding the causes of the infertility. As most people are deeply convinced of the supernatural causes of infertility, it is not surprising that infertile people often patronize traditional healers and religious leaders very early. Orthodox medical practitioners are often consulted later when religious and traditional methods have failed to provide a solution to the infertility.

*“I am using both traditional and orthodox medicine, but I first went to the herbalist”*- (A 38-year old infertile woman in IDI).

The traditional remedies ranges from making sacrifices of animals to pacify the gods or ancestors for wrongdoing to taking of local concoctions prepared from herbs. They may also be requested to perform some rituals at a place where two, three or four roads cross or at where ants live and this was the pronounced ritual. Another ritual that is often practiced is the wearing of prescribed costume mostly by the female partner, which is removed at the entrance to their residents. Another treatment, which was mentioned both in individual interviews and in focus group discussions was the excision of some part of the clitoris, which is believed to cause infertility in women. It is belief that an ‘extra-germination’ of the clitoris was capable of causing infertility in women. The gods are believed to give direction when consulted by the healers.

*“….My wife bled highly when a portion of her clitoris was excised by a traditional medical practitioner as treatment for infertility”*- (A 46-year childless man in IDI).

Churches also prescribed number of days of fasting and prayers and making of some special offerings to the church. References were also made of washing of women genitalia with holy water and anointing oil prepared and blessed by the faith healers.

*“I was given some anointing oil to smear on my private part before having sex with my husband”*- (A 36-year old infertile woman in IDI).

Another important consideration in the choice of a practitioner is the issue of privacy. As infertility is considered a very sensitive issue in the community, people often seek out practitioners who will be able to keep their infertile status a secret. Both individual interviews and Focus-group participants had different views about which practitioners; orthodox, traditional or spiritual, would maintain the most confidentiality. Although it was generally agreed that traditional healers are capable of assuring the most confidentiality, nevertheless, the view was expressed that traditional practitioners often exploit women consequently, either financially or sexually. This did not however reduce the power of the treatment provided by the traditional healers, but was considered “a good price to pay” to get a child to make you happy and save you from ridicule and your marriage. In general, the *modus operandi* of traditional medical practitioners was believed to maintain better privacy than that of biomedical practitioners. Their facilities are often situated at the outskirt of the community where community members hardly go except for those with problems who have gone to consult.

*“Traditional medical practitioners get their powers from divine sources and are therefore compelled to maintain their practice in secret otherwise they loss the powers and this is different from the hospital where nobody cares about privacy”*- (An FGD female participant).

Many participants also recognized the importance of going to the hospital for tests and firmly believed that the doctors can often determine the exact cause of the infertility, and prescribe drugs to treat it. However, the medical approach is often not used immediately since biological factors are not acknowledged as prominent causes of infertility in the community. Many people believe that Western medical treatment can only help if there is a biological cause of infertility. If people believe that the infertility was caused by a curse or spell, or by gods and ancestors, they will seek an appropriate solution, which may not include the biomedical practitioners. They may seek help from traditional doctors or spiritualists first, and come to the medical doctor later. This will be done for additional help or to treat a problem caused by the herbal treatments. The social stigma attached to infertility problems means that people are cautious of revealing their problems, and the hospital environment may be too open to accommodate such secrecy, participant's claim. Participants also described assigning special days and clinics for infertility in hospitals as broadcasting to the whole world about the infertility status of the clients and therefore not confidential enough.

*“You go to hospital, and they will announce that those with infertility should go this consulting room telling everybody of your problem. The next thing you see is that people are pointing fingers at you in town”*- (A female infertile woman in FGD).

Interestingly, either couples with infertility sought remedies individually or the women were the only people who reported to be seeking treatment especially in biomedical facilities. Males also tended to seek for treatment alone at the traditional medical practitioners and only invited their wives when both are to perform some rituals. It was also very interesting to note that when it is even established that the aetiological factors had to do with male factors, traditional medical practitioners still focused on women and they are still compelled to lead the rituals.

*“It is we, the female who are always concerned about childlessness, because of the pressures we get from our in-laws, so we are the ones who look for treatment. Your husband may even refuse when invited by the medical practitioners, even when the cause is from your husband you are still supposed to do something as a ritual”*- (An infertile woman in FGD).

### Prevention of Infertility

The prevention of infertility was also unanimous and education was seen as the core preventive strategy especially for the younger generation as many of the perceived causes are consequences of behaviours during youth. People commonly suggested that drugs used for abortion should be banned even though there were other herbs that were believed to be capable of inducing abortion, which are believed to be relatively safer than medical abortificients.

*The drugs used to cause abortion are sold around by individual in the community but not in the drug stores*- (An FGD female participant).

In several cases, the participants suggested that the use of dangerous drugs should be stopped. It was suggested in some cases that the “doctors” themselves were selling these drugs. When prompted on the people described as “doctors” it was discovered that the term was used to refer to people that sell drugs in the community and various workers in biomedical health facilities. Many of the participants expressed the wish that all abortions should be eliminated. When the issue of prevention turned to the use of contraceptives, respondents were split on the value of contraceptives: while often, people believed that contraceptives could prevent pregnancy, and thus prevent illegal abortion and the infertility that might follow; others felt that contraceptives themselves caused infertility, and that their use should be stopped. Some of these same people felt that women should be encouraged to bear the children if they became pregnant because of the fixed number of children every woman is destined to conceive and give birth.

*“Those drugs that prevent pregnancy should be stopped, they also cause infertility and in this community, many women have complained that they are unable to conceive again after using those drugs”*-(An FGD female participant).

Another preventive strategy of future childlessness was education of the youth to desist from unhealthy lifestyles like excessive alcohol intake, use of sex enhancing drugs and smoking. A call was made on government to place a ban on the importation of such products.

Supernatural causes are regarded as not within the control of the individuals hence difficult to prevent. However, respecting the norms of society and adhering to codes of conduct for marriage couples were central in preventing the social causes of infertility.

*“Respect for societal norms will prevent infraction with the gods and ancestors who are capable of visiting infertility on couples”*- (A male FGD participant).

## Discussion

There was little diversity in the participants' definition of infertility. Many defined it simply as a woman's inability to bear children or inability of couples to have children. Focus-group participants generally recognized the concepts of primary and secondary infertility in addition to another form of infertility, which will be described as “*tertiary infertility”*. The surname is very important in the culture of the people of Northern Ghana and since women who get married are, supposed to name their children after their husband, or use the husband's surname, male children symbolize the worth of the man [25], [26]. Couples without male children can therefore be described as suffering from “*tertiary infertility”* as they are treated the same way as those without children. Primary infertility has been described as the inability of couples to conceive after two years of unprotected regular sex and secondary infertility as the inability of couples who have conceived before to beget children [9]. The desire to have male children poses a threat to family planning services as well as STI control programmes as males generally experiment with other women in an attempt to have male children [25]. The use of children as farm helps also deny children of formal education and a challenge to achieving universal primary education to achieve Millennium Development Goal 2.

Knowledge on the biological causes of infertility was generally high and unanimous. The role of STI as a common cause of infertility for both males and females was undisputed. However, couple infertility was more attributed to female factors. Previous use of contraceptive was the mostly mentioned biological causes of infertility for females. Northern Ghana is a poverty endemic region in the country and therefore efforts are tailored towards reducing the fertility rate of couples. The perception that the use of contraceptives can cause infertility in females therefore poses a challenge to approach adopted by the Ghana Health Service in her reproductive health policy to prevent unwanted pregnancies and ensure good child and maternal health aimed at accelerating progress towards achieving the Millennium Development Goals, 4 and 5 respectively. Family planning providers must also be concerned about sexually transmitted diseases, induced abortion and infertility and they should be able to find ways to incorporate such concerns into their programs. An integrated reproductive health policy that encompasses family planning, prevention of STIs and infertility will give a holistic approach to problem solving in Ghana. Vertical and disease specific approach may undermine and neglect some equally significant social problems in community context.

The belief that Female Genital Mutilation (FGM) can cause infertility in females is of medical importance. FGM is a negative cultural practice that is commonly practiced among people from the northern part of Ghana [25], [27]. The perception that FGM can cause infertility therefore implies that parents will resist the excision of the clitoris of their female children making the cultural practices outdated. At the opposite side of this is the belief that an extra-germination of the clitoris could cause infertility. Female genital mutilation may therefore be embedded in this belief and therefore a major challenge to the prevention of this unhealthy traditional practice. The worldview that violating of marriage code of conduct could result in infertility had some positive effects on marriage unions. Because of the supernatural supervision of marriage by ancestors and sanctioning of couples who violate the codes, married couples live within the societal prescription of the conduct of marriage couples. This creates harmony and prevents extramarital activities especially for female. This may therefore reduce incidence of STIs among couples.

Cigarettes contain nicotine, which has received much attention for its interference with normal endocrine function, it has been shown to cause testicular atrophy, gonadal dysfunction, and male factor infertility by triggering testicular cytotoxicity [28] and this was cited as one of the causes of infertility in males. However, of medical interest is the role that masturbation places in infertility. Though it is unclear how masturbation can cause infertility, the community believed that masturbation was a form of abortion. The concept of male abortion (masturbation) to the best of the knowledge of the authors has never been reported previously, and this serves as a social factor that plays a very important role among communities in Northern Ghana.

The study revealed that couples used three main medical outlets: spiritual, traditional medical practitioners and biomedical. Couples used these facilities concurrently or in sequence. This finding agrees with similar studies in Nigeria and South Africa. Several women with infertility consulted herbalist, apart from a witch doctor. Others visit churches for treatment because they perceive infertility as a misfortune, which God can redress [29]. Traditional health care was also identified as an important alternative source of understanding, coping and medication for health problems, including infertility in the Gambia [30]. The traditional medical practitioners were the most utilized outlet of care as infertility was given a more spiritual cause than medical and they were deemed to provide better privacy than biomedical facilities. Contrary to this finding, medical treatment was the preferred choice for many couples in an earlier study [31]. Confidentiality and ability to keep infertile status of couples as a secret determined the help seeking behaviour. Since many respondents believed that the environment and mode of operation of the biomedical health facilities was not private enough, it was generally not conceived as first line treatment. Traditional medical practitioners in this study are very instrumental in the diagnosing and management of infertility in Northern Ghana. It is therefore very relevant to integrate traditional medical practitioners into the primary health care system because of the multiple advantages it presents. A good collaboration among the traditional and orthodox medical practitioners will provide an opportunity for the training of traditional medical practitioners in current scientific knowledge and enhance inter-practitioner referral system, which can cater for both biological and spiritual aetiologic factors.

However, the use of specialized clinics or designation of some days within a general hospital for some disease conditions is widely used in Ghana, the findings of this study point to the negative effects and perception among community members especially for disease conditions with stigma. The general believe in the community is that such clinics literally broadcast the disease conditions of clients and hence additional measures should be put in place to ensure privacy and confidentiality of clients. Both the use of donated egg and sperm were generally unacceptable among participants in this study. Adoption was viewed as service to God but not a remedy for infertility. A study also found negative feeling associated with adoption in a study of Hindu couples (in India) because it was perceived as highly visible indicator of infertility [32]. Donated eggs seemed to be marginally more acceptable than the use of donated sperm, which is seen as sexual in a way [30].

Some of the couples, who believed that their infertility was due to witchcraft, curse or any cause other than physical are those who sought for spiritual help. Some of the couples had sought help from spiritual churches and prayer camps. The study revealed that traditional medical practitioners prescribed rituals including wearing of some special costumes. In Nigeria, herbalists prescribe certain ritual or actions, such as the women bathing at night at a place where roads meet, or making sacrifices of food to evil spirits that may be causing the problem and leaving the items at the crossroad [4]. The findings of this study are therefore similar to the study in Nigeria. Of medical interest is also excision of some portions of the clitoris by some traditional medical practitioners. Excessive bleeding, scarring and difficulty during labour could result from such procedures. Since the equipment used for such procedures are not usually sterile, this predisposes the victims to infections. Insertion of objects and concoctions into the vagina of women as treatment for infertility can also result in infections that may aggravate the situation.

In is clear from the study that the concept of health have social undertones. Societal perception of a disease directly affects the behaviour of the community to people suffering from that disease and it intends affect the help-seeking behaviour of clients. In a typical pronatalist community like Northern Ghana, the concept of reproductive health can only be meaningful, when opportunities are available for individuals to beget the number of children they prefer. This can only be achieved through an integrated approached to reproductive health. Infertility and childbearing should be given a priority and the possibility of fertility insurance should be explored.
